# Sex differences in patients with symptomatic carotid stenosis included in three observational cohorts

**DOI:** 10.1093/esj/aakag018

**Published:** 2026-04-21

**Authors:** Christine Kremer, Cheryl Carcel, Elias Johansson

**Affiliations:** Neurology Department, Skåne University Hospital, Department of Clinical Sciences Lund University, Lund, Sweden; Brain Health Program, The George Institute for Global Health, University of New South Wales, Sydney, Australia; Wallenberg Center for Molecular Medicine, Umeå University, Umeå, Sweden; Neuroscience and Physiology, Clinical neuroscience, Sahlgrenska Academy, Gothenburg, Sweden

**Keywords:** carotid artery stenosis, carotid endarterectomy, carotid stenting, sex differences

## Abstract

**Introduction:**

Sex differences in symptomatic carotid artery stenosis have been reported with conflicting results, with knowledge gaps regarding the degree of carotid stenosis, management and preoperative ischaemic events. This study aimed to determine sex differences in symptomatic carotid artery stenosis prior to undergoing carotid endarterectomy (CEA) or carotid artery stenting (CAS).

**Patients and methods:**

This was a secondary analysis using pooled data from 3 cohorts with recent symptomatic ≥ 50% carotid artery stenosis undergoing preoperative evaluation for possible CEA or CAS.

**Results:**

The cohorts included 934 participants (mean age 73 years); 299 (32%) were women. Women underwent CEA or CAS less often (66% vs 73%; *P* = .026), which was not significant after adjustment for the degree of stenosis (*P* = .17). The number of carotid near-occlusions (CNOs) was comparable (31% women vs 32% men, *P* = .93). Women without CNO more often had 50%–69% stenosis (67% vs 55%, *P* = .029) and smaller internal carotid artery (ICA) diameters (4.0 mm vs 4.2 mm, *P* < .001). Women with CNO more often had full collapse (42% vs 25%, *P* = .011). The risk of preoperative ischaemic events did not differ.

**Discussion and conclusion:**

There were significant sex differences in the degree of stenosis and anatomy of the ICA, potentially explaining why fewer women underwent CEA/CAS. No sex differences in the risk of preoperative early stroke recurrence were found.

## Graphical Abstract

**Graphical Abstract:**
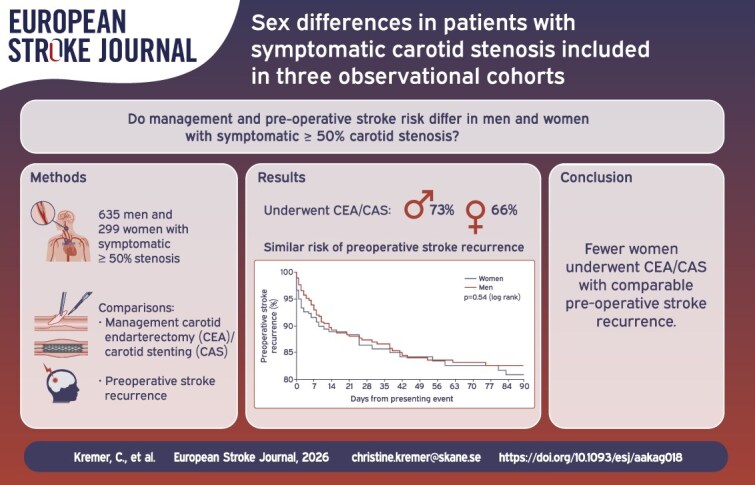

## Introduction

Ischaemic stroke due to atherosclerotic disease accounts for 15%–20% of all ischaemic strokes. In the western population, carotid artery disease is the most common atherosclerotic stroke aetiology, is more common in men and its prevalence increases with age.^1^ Carotid endarterectomy (CEA) or carotid artery stenting (CAS) performed within 2 weeks in patients with symptomatic ≥ 50% carotid artery stenosis is the treatment of choice to prevent further embolisation.^2^

Sex differences in the management of symptomatic carotid disease have been described; however, the results are conflicting. In postmenopausal women, declining oestrogen levels likely contribute to a different pattern of atherosclerosis, arterial stiffness and a higher risk of hypertension and stroke in postmenopausal women.^3^ Differences in plaque morphology have been described, with men showing larger plaques, more necrotic lipid-rich cores, intraplaque haemorrhage and lower plaque stability.^4^ A higher risk of restenosis after CEA has been reported in women.^5^ The relative size (diameter ratio) of the internal carotid artery (ICA) compared to the common carotid artery and external carotid artery (ECA) is smaller in women than in men, indicating a smaller distal ICA in women, although this was only assessed in relative numbers.^6^ Furthermore, anatomical variants, such as twisted carotid bifurcation of the ICA, are more often observed in women, which can add to the perceived technical difficulties during CEA.^7^ Although these studies focus on sex differences in atherosclerotic disease and plaque morphology in carotid artery stenosis, the degree of stenosis among those with symptomatic ≥ 50% stenosis has not been studied yet.

The outcomes are worse in women than in men; women have higher complication rates with both CEA and CAS, although no differences in mortality have been found.^8^ On average, women benefit less from symptomatic CEA in terms of 5-year reduction in ipsilateral ischaemic stroke after considering the perioperative risk.^9^ However, this average is often not representative: among patients treated within 2 weeks of the last event, women seem to have more benefit than men when the degree of stenosis is ≥ 70% without carotid near-occlusion (CNO), whereas the benefit is similar for 50%–69% stenosis.^10^ Beyond 2 weeks, the benefit of CEA seems to be reduced faster in women than in men, especially in cases of ≥ 70% stenosis.^10^ For preoperative early stroke recurrence risk, the main risk factors are the degree of stenosis (mainly the existence of CNO with full collapse) and type of presenting event, whereas sex is not a risk factor.^11-13^ However, no study has performed risk analyses for early recurrence stratified by sex. In addition, we found no studies on sex differences in the selection of revascularisation procedures.

This study aimed to identify sex differences in the management of carotid artery stenosis based on differences in the degree of carotid artery stenosis, early recurrent ischaemic events and selection of revascularisation in 3 well-defined cohorts of patients with symptomatic carotid artery disease.

## Patients and methods

### Participants

This was a secondary analysis using pooled data from 3 cohorts of patients (additional neurological symptoms before surgery of carotid arteries [ANSYSCAP], Transatlantic Carotid Near-Occlusion Study [TACNOS] and Umeå Carotid Cohort [UCC]) with recently symptomatic ≥ 50% carotid artery stenosis (Table 1).^11-13^ The cohorts included participants from a single tertiary centre during a 15-year period (the stroke unit at the University Hospital of Northern Sweden), with each cohort including participants for 2.4–5 years. All participants underwent preoperative assessments and were either local patients or were referred from 11 other hospitals in the region. In all cohorts, patients had to undergo CEA or CAS or be preliminarily eligible for these procedures at the time of presentation. Patients for whom CEA/CAS was never considered were excluded, such as those with very advanced age (often > 90 years), severe comorbidities or major stroke as a presenting event (without clear improvement) that precluded CEA/CAS. One exception was that patients (local or referred) were also included if it was a recurrent stroke after presentation that made them ineligible for assessment. In this analysis, all cases from these clinically selected cohorts were included (no additional exclusion criteria).

**Table 1:** Summary of the cohorts.

**Study work name**	**ANSYSCAP**	**TACNOS**	**UCC**
Site	Umeå, Sweden	Umeå, Sweden	Umeå, Sweden
Period	Aug 2007–Dec 2009	Jan 2010–Dec 2014	Feb 2018–May 2022
Design	Prospective	Retrospective	Prospective
Consecutive series	Yes	Yes	Yes^a^
Main study aim	Prognosis of symptomatic ≥ 50% carotid stenosis	Prognosis of symptomatic carotid near-occlusion	Diagnostics, prognostics and pathophysiology of carotid near-occlusion
Ascertainment	Daily on-site	Search for consecutive CTAs	Daily on-site
Inclusion criteria	Symptomatic ≥ 50% carotid stenosis^b^	Symptomatic ≥ 50% carotid stenosis^b^	Symptomatic ≥ 50% carotid stenosis^b^
Included in this analysis	229	371	334
Modality for degree of stenosis	Ultrasound 98% (*n* = 224) CTA: 22% (*n* = 83) Combined assessment from clinic used	CTA 100% (*n* = 371) Study CTA assessment used	CTA (*n* = 325) Ultrasound alone (*n* = 9) Study CTA assessment used when available
Ethics	Waived^c^	Approved^d^, need of informed consent waived	Approved^d^, informed consent required for inclusion

Abbreviations: ANSYSCAP, additional neurological symptoms before surgery of symptomatic carotid stenosis, a prospective study; TACNOS, transatlantic carotid near-occlusion study; UCC, Umeå carotid cohort.

^a^Had a 6-month break due to COVID-19 in 2020.

^b^Cases with carotid occlusion and asymptomatic stenosis were also assessed, but not relevant for this analysis.

^c^The need for ethics approval was waived by the regional ethics board in Umeå. This was a standard practice at the time, but changed by revision of Swedish ethics laws in 2008.

^d^The study was approved by the regional ethics board in Umeå.

### Data acquisition

All parameters were recorded in a similar manner for each cohort. All recurrent events (stroke, retinal artery occlusion [RAO], TIA and amaurosis fugax [AFX]) were assessed in detail through patient interviews and review of medical records. The source data for all recurrent strokes and RAOs were rechecked by E.J. for consistency in 2022 after the final inclusion in all cohorts. The degree of stenosis was based on ultrasound in the first cohort, exclusively on CTA in the second and primarily on CTA in the third (with ultrasound used when CTA was not possible). The degree of stenosis was assessed using the North American Symptomatic Carotid Surgery Trial (NASCET) criteria.^9^ The separation of CNO and stenoses without CNO was performed with feature interpretation assessments of CTA in 2 of the 3 cohorts, as presented elsewhere when CNO data were presented for these cohorts.^12,13^ In the third cohort, this was not possible because CTA was not widely available at that time. In short, 2 blinded observers assessed whether the distal ICA diameter was reduced and whether stenosis was the most reasonable cause. The features used to make this determination were stenosis severity, distal ICA diameter in mm, and relative to the contralateral ICA (ICA:ICA ratio) and the ipsilateral ECA (ICA:ECA ratio); however, anatomical variation in the Circle of Willis and possible distal disease were also considered.^12,13^ The separation of CNO with and without full collapse was performed using the 2022 Johansson criteria (≤2.0 mm distal ICA diameter and/or ≤ 0.42 side-to-side ICA diameter ratio).^14^ In cases where only ultrasound was available, CNO with full collapse was diagnosed in cases with severe ICA stenosis with a very low stenosis velocity.^15,16^ There were no criteria for CNO without full collapse on ultrasonography.

### Outcomes and definitions

No primary outcome was defined as the aim was to make a comprehensive assessment, making assessments of baseline differences, management choices and recurrent events equally valid. Recurrent events combined ipsilateral ischaemic stroke and ipsilateral RAO, similar to the pooled analysis of NASCET and the European Carotid Surgery Trial (ECST),^9^ but separately also combined stroke, RAO, TIA and AFX (all ipsilateral and ischaemic).

For consistency with former research, stroke was separated from TIA based on duration (less than or equal to 24 h vs > 24 h), and thus TIA with signs of new ischaemia on radiology was not considered a stroke. Similarly, the RAO was separated from the AFX using the same time window criteria. Symptomatic stenosis (a main inclusion criterion) was defined as a stenosis with a relevant ipsilateral ischaemic event within the last 6 months, ie, an event that fitted the distribution area of the stenosis. Other simultaneous causes (such as atrial fibrillation) were accepted as long as the case as a whole (clinic and radiology) was not obviously from another source (such as recent events/ischaemia in multiple territories not explained by anatomical variants). The presenting event was the last event that occurred before the patient sought medical care.

### Ethical considerations

All 3 cohorts passed the requisite ethics review by the regional ethics board in Umeå, Sweden, with slight differences between the cohorts (Table 1).

### Analyses and statistics

Categorical variables are presented with absolute and relative frequencies and were compared using the 2-sided χ^2^-test or Mann–Whitney *U* test (when ordinal). Continuous data were considered parametric if skewness and kurtosis were between −1 and + 1. Parametric variables are presented as mean and SD and were compared using the *t*-test. Non-parametric variables are presented as medians with IQRs, compared using the Mann–Whitney test.

Two relevant outcomes (referral and selection for CEA/CAS) were subjected to multivariable analyses (binary logistic regression). Potential confounders (covariates associated [*P* < .10] with either sex [at baseline] or the outcome) were entered into the model if the number of missing data points was < 20. To further augment the understanding of individual confounders, sex was adjusted for each significant factor in the multivariable model, including the interaction test. A similar analysis was not undertaken for the 30-day risk of perioperative stroke or death, as previously described.^17^

Event outcomes were assessed using Kaplan–Meier analyses, where death, 90 days after the index event, and CEA/CAS procedures were used as censors. The presenting event was an index event. No loss of follow-up occurred during the study period. Group comparisons were performed using the log-rank test. These analyses were stratified by degree of stenosis and type of presenting event (stroke and TIA combined vs retinal), as these factors are known to affect the risk.^11-13^ Cox regression was used to adjust for sex, degree of stenosis, type of presenting event and to assess the interaction.

Statistical significance was set at *P* < .05. IBM SPSS 28.0 was used for the calculations.

## Results

We included 934 participants with symptomatic carotid stenosis ≥ 50%. Of these, 299 (32%) were women. Compared with men, women were less likely to have a history of stroke, revascularisation and statin treatment, more likely to have current symptomatic peripheral artery disease and to be current smokers and had higher median cholesterol and high-density lipoprotein levels (Table 2). However, there were no differences between the sexes in the underlying study (ie, over time), mean age, delay in presentation or type of presenting event.

**Table 2:** Baseline characteristics.

		**Women (*n* = 299)**	**Men (*n* = 635)**	***P* value**
Study	ANSYSCAP *n* (%)	83 (36)^a^	146 (64)^a^	.26
	TACNOS *n* (%)	111 (30)^a^	260 (70)^a^	
	UCC *n* (%)	105 (31)^a^	229 (69)^a^	
Age mean (SD)		72.3 (8.1)	72.7 (7.7)	.47
Previous stroke *n* (%)		32 (11)	100 (16)	.044
Current angina *n* (%)		36 (12)	91 (14)	.36
Previous myocardial infarction *n* (%)		53 (18)	124 (20)	.53
Heart failure *n* (%)		11 (4)	22 (5)	.31
Atrial fibrillation *n* (%)		31 (10)	72 (11)	.74
Current symptomatic peripheral artery disease *n* (%)		42 (14)	52 (8)	.007
Previous arterial revascularisation *n* (%)		63 (21)	176 (28)	.030
Current smoking *n* (%)		73 (25)	94 (15)	<.001
Diabetes *n* (%)		73 (24)	171 (27)	.50
Hypertension *n* (%)		272 (91)	576 (91)	.91
Total cholesterol mmol/L median (IQR)		4.6 (3.9–5.7)	4.3 (3.6–5.2)	<.001
LDL cholesterol mmol/L median (IQR)		2.4 (1.8–3.4)	2.3 (1.8–3.2)	.15
HDL cholesterol mmol/L median (IQR)		1.3 (1.1–1.5)	1.1 (0.9–1.3)	<.001
Sought health care on the day of presenting event *n* (%)		197 (66)	442 (70)	.26
Type of presenting event	AFX *n* (%)	48 (16)	93 (15)	.96
	RAO *n* (%)	14 (5)	30 (5)	
	TIA *n* (%)	98 (33)	210 (33)	
	Stroke *n* (%)	139 (47)	302 (48)	
Right side symptomatic *n* (%)		139 (47)	319 (50)	.29
Prior medical treatment	No AP/AC *n* (%)	153 (53)	282 (46)	.12
	SAPT *n* (%)	115 (40)	258 (42)	
	DAPT *n* (%)	3 (1)	11 (2)	
	AC *n* (%)	19 (7)	63 (10)	
	Blood pressure lowering *n* (%)	232 (80)	475 (77)	.34
	Statin *n* (%)	126 (44)	311 (51)	.063
Degree of ipsilateral stenosis^b^	50%–69% *n* (%)	98 (46)	181 (37)	.004^c^
	≥70% without CNO *n* (%)	48 (23)	150 (31)	
	CNO without full collapse *n* (%)	38 (18)	115 (24)	
	CNO with full collapse *n* (%)	28 (13)	38 (8)	

Abbreviations: AFX, amaurosis fugax; AP, antiplatelet therapy; AC, anticoagulation therapy; CNO, carotid near-occlusion; DAPT, dual antiplatelet therapy; HDL, high-density lipoprotein; LDL, low-density lipoprotein; RAO; retinal artery occlusion; SAPT, single antiplatelet therapy.

^a^Percentages presented per study instead of per sex, for easier interpretation.

^b^To achieve a uniform grading, this only includes cases with CTA data, ie, excluding all cases from ANSYSCAP and 9 cases from UCC with CTA contraindications. The 238 excluded cases relied foremost on ultrasound grading with poor CNO detection. Beyond that, the excluded cases had a similar sex difference in degree of stenosis (*P* = .032; Table S1).

^c^Post-hoc assessments between men and women: CNO vs not CNO: *P* = .93; 50%–69% CNO vs ≥ 70% without CNO: *P* = .029; CNO with full collapse vs CNO without full collapse: *P* = .011.

### Degree of stenosis

There were sex differences in the degree of stenosis (Table 2). Among ≥ 50% stenoses without CNO, women more often had 50%–69% stenosis than men (67% vs 55%, *P* = .029). There were no sex differences in the rate of CNO (31% women, 32% men, *P* = .93), but among CNOs, women more often had full collapse (42% vs 25%, *P* = .011; Table 2). In the whole cohort, there was no difference in stenosis diameter and ICA:ICA ratio, but women had smaller distal ICAs (median 3.6 mm vs 3.9 mm, *P* < .001) and ICA:ECA ratios than men (Table S1). This pattern was also observed for stenoses without CNO; however, for CNOs, both the distal ICA diameter and ICA:ICA ratio were smaller in women than in men, but not the ICA:ECA ratio.

### Management

Among referred patients, the share of women (30%) was lower than the share of local patients (38%, *P* = .043; Table 3). When adjusting for potential confounders, this was borderline significant (*P* = .0503) but significant when adjusting only for likely confounders (*P* = .025; Table S2). When adjusting for each significant factor in the multivariable model one-by-one, sex always remained significant (*P* range .029–.039), and there were no significant interactions (Table S3).

**Table 3:** Sex differences in management.

		**Women (*n* = 299)**	**Men (*n* = 635)**	***P* value**
**Pre-revascularisation**				
Referred from other hospital *n* (%)^a^		228 (76)	521 (82)	.043
Medical therapy during study period^c,b^	No AP/AC *n* (%)	3 (2)	4 (1)	.38
	SAPT *n* (%)	128 (68)	230 (62)	
	DAPT *n* (%)	36 (19)	84 (23)	
	AC *n* (%)	21 (11)	56 (15)	
	Blood pressure lowering *n* (%)	164 (90)	333 (90)	.88
	Statin *n* (%)	170 (92)	348 (94)	.48
Thrombolysis for presenting event *n* (%)		19 (6)	46 (7)	.68
**Revascularisation**				
Revascularisation with CEA or CAS *n* (%)		196 (66)	463 (73)	.026
Revascularisation type: CEA *n* (%)		185 (94)	430 (93)	.18
Days between presenting event and revascularisation median (IQR)		12 (7–29)	12 (7–27)	.91
Main reason for not undergoing revascularisation	Major stroke recurrence *n* (%)	7 (7)	12 (7)	1
	Technical issues *n* (%)	11 (11)	22 (13)	.7
	Patient refusal *n* (%)	8 (8)	16 (9)	.83
	Unfavourable risk/benefit *n* (%)	22 (21)	24 (14)	.13
	Comorbidities *n* (%)	4 (4)	9 (5)	.77
	Low perceived benefit *n* (%)	25 (24)	26 (15)	.077
	Perceived to be < 50% *n* (%)^a^	19 (18)	43 (25)	.24
	Perceived to be occluded *n* (%)^a^	4 (4)	12 (7)	.43
	Obvious mismanagement *n* (%)^b^	3 (3)	8 (5)	.55
30-day stroke/death after revascularisation *n* (%)		16 (8)	15 (3)	.009

^a^Conversely, among 749 referred patients, 228 (30%) were women, whereas among 185 local patients, 71 (38%) were women. The referred participants underwent CEA or CAS more often (73%) than local participants (60%, *P* < .001). Regarding causes to not perform CEA or CAS, only a weak tendency was seen for more often perceived low benefit in referred participants (21%) than local participants (12%, *P* = .11), with all other causes being similar (*P* ≥ .33).

^b^Not collected in TACNOS (*n* = 371).

^c^Applicable to TACNOS and UCC where degree of degree of stenosis used in the study was set be reassessing CTAs (done after clinical management had occurred). Thus, cases can have a ≥ 50% stenosis according to study assessment but not undergo revascularisation as they were perceived to have < 50% stenosis or occlusion in the clinic.

^d^Cases detected in retrospect during TACNOS and UCC. Refers to obvious misinterpretations of clinical data or overlooking to take obviously warranted action when stenosis was detected (such reports left unsigned or signed with significant delay and prompted no action).

Abbreviations: AC, anticoagulation therapy; AP, antiplatelet therapy; CAS, carotid artery stenting; CEA, carotid endarterectomy; DAPT, dual antiplatelet therapy; SAPT, single antiplatelet therapy; TACNOS, transatlantic carotid near-occlusion study.

Both men and women received similar medical therapy, were treated with thrombolysis similarly often and had similar delays in revascularisation (Table 3). However, women underwent revascularisation less often (66%) than men (73%, *P* = .026; Table 3). Among local patients, no sex difference was observed (58% vs 61%, *P* = .76); this sex difference only existed among referred patients (68% vs 76%, *P* = .032). The main reasons for not undergoing revascularisation were recorded, and no single reason was more prevalent in women than in men; however, there was a tendency for more women to be deferred because of an unfavourable risk/benefit ratio (ie, clearly increased risk or the combination of moderately increased risk and lowered benefit) or low perceived benefit (Table 3). Low perceived benefit means that the long-term net risk reduction with revascularisation was deemed so low that the procedure was not beneficial for the patient (ie, without a risk increase for the procedure above normal). In multivariable analyses, the sex difference in the selection for revascularisation was no longer significant (*P* =0.17; Table S4). When adjusting for each significant factor in the multivariable model one-by-one, sex became non-significant when adjusted for degree of stenosis (*P* = .22) but remained significant when adjusted for all other factors (*P* range .010–.039), and there were no significant interactions (Table S5).

### Recurrent events

**Figure 1:**
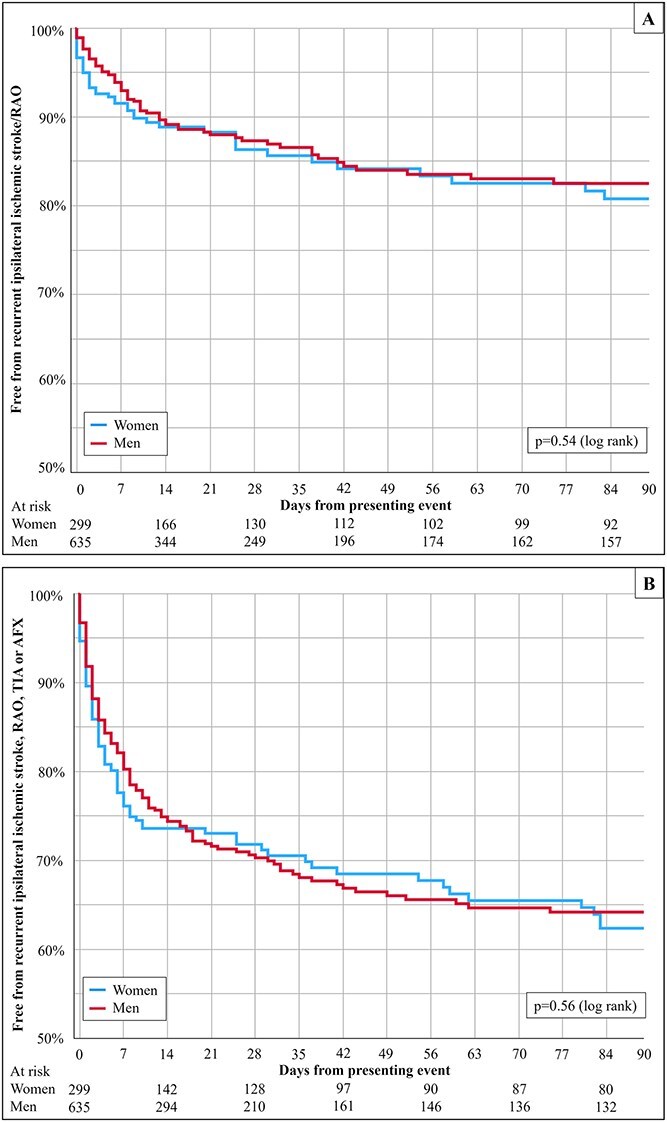
Kaplan-Meier analyses of the preoperative risk of (A) recurrent ipsilateral ischemic stroke or retinal artery occlusion and (B) recurrent ipsilateral ischemic stroke, retinal artery occlusion, TIA, or AFX.

There were no sex differences in the risk of preoperative ipsilateral ischaemic stroke/RAO or ipsilateral ischaemic events (Figure 1).

The risk of preoperative ipsilateral ischemic stroke/RAO, unadjusted hazard ratio (HR) for sex was 0.9 (95% CI, 0.6–1.3; *P* = .54), which was unaffected when adjusting for the degree of stenosis and presenting event (adjusted HR 0.9; 95% CI, 0.6–1.3; *P* = .48) and without interaction between sex and degree of stenosis (*P* = .79) or sex and presenting event (*P* = .87). When stratified by the degree of stenosis, there were no sex differences in the risk of preoperative ipsilateral ischaemic stroke/RAO (Figure S1). Conversely, the risk of preoperative ipsilateral ischaemic stroke/RAO was significantly affected by the degree of stenosis (higher risk in CNO with full collapse than in all 3 other groups with similar risk) when assessed separately in women (*P* = .015) and men (*P* = .029). When stratified by the type of presenting event, there were no sex differences in the risk of preoperative ipsilateral ischaemic stroke/RAO (Figure S2). Conversely, the risk of preoperative ipsilateral ischaemic stroke/RAO was significantly affected by the type of presenting event (higher risk in stroke and TIA than retinal events) when assessed separately in women (*P* = .02) and men (*P* < .001).

Women had a higher perioperative risk (8%) than men (3%, *P* = .009; Table 3), as previously reported for these cohorts.^17^

## Discussion

In this large observational study, several sex differences in the management and grading of carotid stenosis were observed.

It seems like women were less often referred for evaluation for CEA/CAS than men; among those referred, 30% were women, whereas among local patients, 38% were women. This did not seem to be caused by confounding, as adjustment for potential confounders had only a minor impact on the results. The selection stage was relevant, as the referred participants underwent revascularisation more often than local patients (OR 1.9, *P* < .001; Table S4). This was expected, as referred participants had already undergone an initial clinical selection for CEA/CAS prior to study enrollment. While a similar selection was sought for local patients, this was not easy to achieve as “would this case have been referred” was not an assessment that is performed. Rather, this selection was applied to cases where CEA/CAS was rejected as an alternative without further consideration, which seems to not have been the same selection as for referring. However, if this selection by referral that caused fewer women to be assessed was clinically reasonable, that is, it was better at removing non-relevant candidates from consideration early, the sex difference at the next stage (undergoing revascularisation) should have been smaller in referred cases than in local cases. Instead, we found a larger difference in those referred, albeit only a numerical difference, as the interaction was negative (*P* = .46; Table S5). Before more definitive conclusions can be drawn, 3 aspects should be considered. First, there was “only” borderline significance in one model and a slightly lower *P*-value in another, very similar model. In other words, independence from confounding factors was not robust. Second, we compared local patients with those referred, assuming that local and referred patients had similar characteristics. While this is a reasonable assumption, it would have been better to compare those referred and not referred from local hospitals; however, such data were unavailable. Third, to the best of our knowledge, this is the first assessment of its kind and a secondary analysis of previously published cohorts; hence, it is hypothesis-generating.

Women undergo revascularisation less frequently than men. According to our data, a lower perceived benefit and higher risk are likely contributing factors. Sex was not significantly associated with the revascularisation rate in the multivariable analysis; however, the underlying study and several other clinical factors were. Of these, the degree of stenosis alone could explain sex differences. Women had a lower grade of stenosis (50%–69% and CNO with full collapse) and consequently a lower probability of revascularisation. These findings are novel resulting from secondary analysis and would need validation in other cohorts before firm conclusions can be drawn. While large carotid stenosis datasets are available, most have not assessed patients with symptomatic carotid artery stenosis who did not undergo revascularisation.

The fact that women have a lower grade of carotid stenosis is consistent with data from a recent meta-analysis observing a lower plaque burden in women.^1^ However, among patients without CNO (where the stenosis should not affect the distal ICA diameter), women had smaller ICA diameters than men. Thus, for a given residual lumen in the stenosis, the degree of stenosis decreased in women when the distal ICA was used as the denominator. The fact that women had smaller ICAs was statistically significant in our cohort (*P* < .001) and is in line with previous assessments that only used relative measurements^6^ and can be considered robust. It is unclear whether the absolute measurements of plaque burden and residual lumen or the relative measurement of the degree of stenosis are the best markers of prognosis. If it is an absolute measurement, the sex difference in the denominator (distal ICA) leads to a general underestimation of the risk in women and an overestimation in men. Conversely, if the currently used relative measurement is the best prognostic marker, that women on average have a lower degree of stenosis than men given the same absolute stenosis severity is not a problem—the prognosis will be equally correct for both sexes. However, the variance between individuals was much greater than the sex difference; therefore, this would be an issue for both men and women with unusually small or large ICAs.

Full collapse of the CNO was significantly more frequent in women. This has not been previously demonstrated and can potentially lead to improved risk stratification and a more individualised approach to the management of symptomatic carotid stenosis in both men and women. The diagnostic criteria for full collapse can be either absolute (distal ICA ≤ 2.0 mm) or relative (ICA:ICA ratio ≤ 0.42).^18^ These are often seen (or are absent) in combination, but the absolute criterion alone was more common in women (*n* = 5) than in men (*n* = 2). This is likely due to their generally smaller distal ICAs compared to men. However, the absolute criterion does not seem to require a revision based on sex. These criteria were derived to detect a high risk of preoperative stroke recurrence in those with full collapse, and 40% (2/5) of the women classified by the absolute criteria alone had an early stroke recurrence (ie, a very high risk).

Preoperative stroke rates were mainly driven by the degree of stenosis and type of presenting event, and no sex differences were observed in these rates. As previously reported, women had a higher perioperative stroke/death risk at 30 days.^17^ The different risk profiles in the management of symptomatic carotid artery stenosis in men and women have been subject to research before, mostly reporting increased peri-interventional and perioperative risk in CAS and CEA.^19^ Since large meta-analysis could not confirm substantial sex differences in the risks and benefits of surgical treatment for symptomatic carotid artery stenosis,^8^ a higher frailty, perceived difficulties, the higher perioperative risk and morbidity could potentially lead to relative undertreatment of women. Recent guidelines recommend carotid surgery within 14 days for symptomatic > 50% carotid artery stenosis, independent of sex, as there were consistent data regarding benefit for both men and women, and lack of association by sex for the risk of surgery.^2,20^ While we show lower revascularisation rate among women, this is not clear evidence of relative undertreatment of women as this association seemed to have been caused by a difference in degree of stenosis. Due to a relative under-presentation of women at advanced age, concomitant risk profile and higher frailty in carotid surgery trials more individualised approach could be adapted in the meantime.^21^ In the future, as the number of women and older women in relevant trials increases, more evidence will help shape the treatment for both sexes.

This study had some limitations. First, it was a single-centre study; therefore, the sex differences observed for grading stenosis and management decisions might not be applicable to other settings. Second, CTAs from ANSYSCAP were not reassessed for artery measurements as these were only stored in thick reformats (3 mm), and only one-third of cases in ANSYSCAP underwent CTA. Hence, the artery measurements were based only on the 2 later cohorts (TACNOS and UCC). Third, the number of patients in some subgroups was too small to allow definite conclusions. Fourth, the multivariable analyses were adjusted for co-variates that were available. Other potentially relevant patient aspects such as frailty might have changed analysis outcomes.

## Conclusion

In conclusion, women had smaller distal ICAs than men and more often had 50%–69% stenosis and CNO with full collapse, potentially explaining why fewer women received CEA/CAS, while no sex differences in the risk of preoperative early stroke recurrence were found.

In the future, more women should be included in RCTs to generate more robust data on both medical and interventional treatment arms to explain the differences found. A more individualised approach, including anatomical differences, frailty and response to medical treatment, should be considered.

## Supplementary Material

aakag018_supplementary

## Data Availability

The research data are available upon reasonable request.
