# Supplementary material for: Sex differences in patients with symptomatic carotid stenosis included in three observational cohorts
Source: Eur Stroke J. 2026 Apr 21;11(4):aakag018. doi: 10.1093/esj/aakag018 (PMC13131239; doi:10.1093/esj/aakag018)

|  | Women (n=299) | Men (n=635) | *p* value |
| --- | --- | --- | --- |
| **Degree of ipsilateral stenosis in all cohorts (mixed exam modalities)** | | | |
| 50-69% n (%) | 128 (43) | 210 (33) | 0.002^a^ |
| ≥70% without CNO n (%) | 98 (33) | 257 (41) |  |
| CNO without full collapse n (%) | 42 (14) | 124 (20) |  |
| CNO with full collapse n (%) | 31 (10) | 44 (7) |  |
| **Degree of ipsilateral stenosis in cases without CTA data** | | | |
| 50-69% % (n/N) | 35 (30/87) | 19 (29/151) | 0.032 |
| ≥70% without CNO % (n/N) | 58 (50/87) | 71 (107/151) |  |
| CNO % (n/N) | 8 (7/87) | 10 (15/151) |  |
| **CTA measurements in all participants** | | | |
| Stenosis diameter, mm median (IQR)^a^ | 1.1 (0.8-1.6) | 1.0 (0.7-1.5) | 0.58^d^ |
| Distal ICA diameter, mm median (IQR) | 3.6 (2.9-4.1) | 3.9 (3.3-4.4) | <0.001^d^ |
| ICA:ICA ratio median (IQR)^b^ | 0.91 (0.69-1.03) | 0.92 (0.72-1.00) | 0.96^d^ |
| ICA:ECA ratio median (IQR) | 1.60 (1.11-1.89) | 1.36 (1.08-1.66) | <0.001^d^ |
| **CTA measurements in stenoses without CNO** | | | |
| Stenosis diameter, mm median (IQR)^a^ | 1.3 (1.1-1.7) | 1.3 (1.0-1.7) | 0.58^d^ |
| Distal ICA diameter, mm median (IQR) | 4.0 (3.6-4.4) | 4.2 (3.8-4.7) | <0.001^d^ |
| ICA:ICA ratio median (IQR)^b^ | 1.00 (0.89-1.05) | 0.98 (0.90-1.03) | 0.18^d^ |
| ICA:ECA ratio median (IQR) | 1.76 (1.48-2.00) | 1.52 (1.32-1.78) | <0.001^d^ |
| **CTA measurement and assessment in CNOs** | | | |
| Stenosis diameter, mm median (IQR)^a^ | 0.5 (0.5-0.8) | 0.5 (0.5-0.8) | 0.34^d^ |
| Distal ICA diameter, mm median (IQR) | 2.4 (1.2-3.0) | 2.8 (2.0-3.3) | <0.001^d^ |
| ICA:ICA ratio median (IQR)^b^ | 0.55 (0.28-0.68) | 0.65 (0.42-0.74) | 0.012^d^ |
| ICA:ECA ratio median (IQR) | 0.96 (0.49-1.10) | 0.97 (0.68-1.11) | 0.44^d^ |
| CNO with full collapse due to ≤2.0 mm distal ICA n (%)^c^ | 5 (7) | 2 (1) | 0.028^a^ |
| CNO: Carotid near-occlusion. CTA: Computed tomography angiography. ECA: External Carotid Artery. ICA: Internal Carotid Artery. IQR: Inter-quartile range.  ^a^ Missing 33 (21 without CNO, 12 CNO) due to extensive calcification in the stenosis. Among stenoses without CNO, these are assigned to be 50-69%.  ^b^ Missing 23 (17 without CNO, 6 CNO) due to contralateral occlusion  ^c^ Distal ICA ≤2.0 mm but ICA:ICA-ratio >0.42. Denominator is all CNOs with full collapse. Of these five women, two suffered a recurrent ipsilateral ischemic stroke on day 0 and 3 after presenting event. Neither of the two men suffered a recurrent ipsilateral ischemic stroke. | | | |

Supplemental table 1. Sex differences in degree of stenosis and artery diameters

|  | | Bivariable | | Multivariable | |  |
| --- | --- | --- | --- | --- | --- | --- |
|  |  | Odds ratio | p | Odds ratio | p |  |
| Women | | 0.7 (0.5-1.0) | 0.039 | 0.7 (0.5-1.0)^a^ | 0.0503^a^ |  |
| Study: ANSYSCAP | | Ref | - | Not used^b^ | - |  |
| Study: TACNOS | | 0.7 (0.5-1.1) | 0.14 |  |  |  |
| Study: UCC | | 1.3 (0.8-1.9) | 0.37 |  |  |  |
| Age (per 10-year increment) | | 0.8 (0.6-0.9) | 0.011 | 0.8 (0.6-1.0) | 0.033 |  |
| Previous stroke^c^ | | 1.2 (0.7-1.9) | 0.46 | 1.3 (0.8-2.1) | 0.38 |  |
| Current angina | | 0.6 (0.4-0.9) | 0.020 | 0.6 (0.4-1.0) | 0.047 |  |
| Previous myocardial infarction | | 1.0 (0.6-1.4) | 0.84 | Not used^b^ | - |  |
| Heart failure | | 0.4 (0.2-0.7) | 0.002 | 0.4 (0.2-0.8) | 0.008 |  |
| Atrial fibrillation | | 0.7 (0.5-1.2) | 0.23 | Not used^b^ | - |  |
| Current symptomatic peripheral artery disease^c^ | | 0.7 (0.4-1.2) | 0.23 | 0.7 (0.4-1.2) | 0.24 |  |
| Previous arterial revascularization^c^ | | 1.0 (0.7-1.5) | 0.93 | 1.3 (0.8-2.1) | 0.27 |  |
| Current smoking^c^ | | 1.3 (0.9-2.1) | 0.19 | 1.3 (0.8-2.1) | 0.27 |  |
| Diabetes | | 1.1 (0.8-1.6) | 0.53 | Not used^b^ | - |  |
| Hypertension | | 0.4 (0.2-0.9) | 0.026 | 0.4 (0.2-0.9) | 0.021 |  |
| Degree of ipsilateral stenosis | 50-69% conventional^a^ | Ref | - | Ref | - |  |
|  | ≥70% conventional^a^ | 1.4 (0.9-2.0) | 0.099 | 1.3 (0.9-1.9) | 0.23 |  |
|  | CNO without full collapse^a^ | 1.4 (0.9-2.3) | 0.14 | 1.2 (0.7-2.0) | 0.49 |  |
|  | CNO with full collapse^a^ | 0.9 (0.5-1.5) | 0.60 | 0.8 (0.4-1.4) | 0.43 |  |
| Type of presenting event | AFX | Ref | - | Not used^b^ | - |  |
|  | RAO | 1.0 (0.4-2.2) | 0.92 |  |  |  |
|  | TIA | 1.1 (0.7-1.8) | 0.65 |  |  |  |
|  | Stroke | 1.2 (0.8-2.0) | 0.38 |  |  |  |
| AFX: Amaurosis fugax. ANSYSCAP: Additional neurological symptoms before surgery of symptomatic carotid stenosis, a prospective study. CNO: Carotid near-occlusion. RAO: Retinal artery occlusion. TACNOS: Transatlantic carotid near-occlusion study. TIA: Transient ischemic attack. UCC: Umeå Carotid Cohort.  ^a^ When adjusting sex only for likely confounders (factors with p<0.05 in the bivariable analysis, all of which remained significant in the multivariable model), adjusted odds ratio was 0.7 (0.5-1.0), p=0.025.  ^b^ Not included as p>0.1 in baseline and bivariable analysis.  ^c^ Included in multivariable assessment due to association (p<0.1) seen in baseline assessment. | | | | | | |

Supplemental Table 2. Bi- and multivariable analysis of if the participants were referred from another hospital, i.e. comparing variables between those referred and those that were local. A low odds ratio indicates fewer in the referred group.

Supplemental Table 3. Explorative multivariable analyses of the participants were referred from another hospital, adjusting for sex for each variable positive in the main multivariable model (Supplemental Table 1) one at a time, and assessing possible interactions.

|  | Sex when adjusted for variable | | Variable when adjusted for sex | | Interaction |  |
| --- | --- | --- | --- | --- | --- | --- |
|  | Odds ratio | p | Odds ratio | p | p |  |
| Age (per 10-year increment) | 0.7 (0.5-1.0) | 0.033 | 0.8 (0.6-0.9) | 0.009 | 0.86 |  |
| Current angina | 0.7 (0.5-1.0) | 0.032 | 0.6 (0.4-0.9) | 0.016 | 0.38 |  |
| Heart failure | 0.7 (0.5-1.0) | 0.029 | 0.4 (0.2-0.7) | 0.001 | 0.99 |  |
| Hypertension | 0.7 (0.5-1.0) | 0.039 | 0.4 (0.2-0.9) | 0.026 | 0.73 |  |
| AFX: Amaurosis fugax. ANSYSCAP: Additional neurological symptoms before surgery of symptomatic carotid stenosis, a prospective study. CNO: Carotid near-occlusion. RAO: Retinal artery occlusion. TACNOS: Transatlantic carotid near-occlusion study. TIA: Transient ischemic attack. UCC: Umeå Carotid Cohort.  ^a^ Included due to association (p<0.1) seen in baseline assessment.  ^b^ Not p<0.1 in baseline (association with sex) and/or bivariable analysis. | | | | | | |

Supplemental Table 4. Bi- and multivariable analysis of selection to revascularization.

|  | | Bivariable | | Multivariable | |  |
| --- | --- | --- | --- | --- | --- | --- |
|  |  | Odds ratio | p | Odds ratio | p |  |
| Women | | 0.7 (0.5-1.0) | 0.022 | 0.8 (0.5-1.1) | 0.12 |  |
| Study: ANSYSCAP | | Ref | - | Ref | - |  |
| Study: TACNOS | | 0.4 (0.3-0.6) | <0.001 | 0.5 (0.3-0.8) | 0.007 |  |
| Study: UCC | | 0.8 (0.5-1.1) | 0.19 | 1.4 (0.8-2.3) | 0.20 |  |
| Age (per 10-year increment) | | 0.6 (0.5-0.8) | <0.001 | 0.5 (0.4-0.7) | <0.001 |  |
| Previous stroke^a^ | | 0.8 (0.5-1.2) | 0.30 | 0.8 (0.5-1.2) | 0.29 |  |
| Current angina | | 0.6 (0.4-0.9) | 0.015 | 0.7 (0.5-1.2) | 0.21 |  |
| Previous myocardial infarction | | 0.8 (0.6-1.1) | 0.21 | Not used^b^ | - |  |
| Heart failure | | 0.4 (0.2-0.7) | 0.003 | 1.0 (0.5-2.1) | 0.97 |  |
| Atrial fibrillation | | 0.5 (0.4-0.8) | 0.004 | 0.6 (0.4-1.0) | 0.047 |  |
| Current symptomatic peripheral artery disease^a^ | | 0.9 (0.6-1.4) | 0.58 | 1.0 (0.6-1.8) | 0.99 |  |
| Previous arterial revascularization^a^ | | 0.8 (0.6-1.1) | 0.26 | 0.8 (0.5-1.2) | 0.25 |  |
| Current smoking^a^ | | 0.9 (0.6-1.2) | 0.42 | 0.7 (0.4-1.1) | 0.10 |  |
| Diabetes | | 0.6 (0.4-0.8) | <0.001 | 0.6 (0.4-0.9) | 0.015 |  |
| Hypertension | | 0.4 (0.2-0.8) | 0.006 | 0.6 (0.4-0.9) | 0.015 |  |
| Referred from other hospital | | 1.9 (1.3-2.6) | <0.001 | 1.5 (1.0-2.3) | 0.020 |  |
| Degree of ipsilateral stenosis | 50-69% conventional^c^ | Ref | - | Ref | - |  |
|  | ≥70% conventional^c^ | 3.0 (2.1-4.3) | <0.001 | 3.1 (2.1-4.7) | <0.001 |  |
|  | CNO without full collapse^c^ | 2.3 (1.5-3.5) | <0.001 | 1.7 (1.1-2.7) | 0.030 |  |
|  | CNO with full collapse^c^ | 0.3 (0.2-0.5) | <0.001 | 0.2 (0.1-0.4) | <0.001 |  |
| Type of presenting event | AFX | Ref | - | Ref | - |  |
|  | RAO | 0.8 (0.4-1.8) | 0.61 | 1.0 (0.4-2.5) | 0.95 |  |
|  | TIA | 0.7 (0.4-1.1) | 0.13 | 0.9 (0.5-1.6) | 0.75 |  |
|  | Stroke | 0.4 (0.3-0.7) | <0.001 | 0.5 (0.3-0.9) | 0.016 |  |
| AFX: Amaurosis fugax. ANSYSCAP: Additional neurological symptoms before surgery of symptomatic carotid stenosis, a prospective study. CNO: Carotid near-occlusion. RAO: Retinal artery occlusion. TACNOS: Transatlantic carotid near-occlusion study. TIA: Transient ischemic attack. UCC: Umeå Carotid Cohort.  ^a^ Included in multivariable assessment due to association (p<0.1) seen in baseline assessment.  ^b^ Not included as p>0.1 in baseline and bivariable analysis.  ^c^ Includes all cases (regardless of modality). If the model was limited to cases with CTA data, adjusted OR for sex was virtually unchanged at 0.8 (95%CI 0.6-1.2, p=0.38), and degrees of stenosis were similarly virtually unchanged. | | | | | | |

Supplemental Table 5. Explorative multivariable analyses of selection to revascularization, adjusting sex for each variable positive in the main multivariable model (Supplemental table 3) one at the time, and assessing possible interaction.

|  | | Sex when adjusted for variable | | Variable when adjusted for sex | | Interaction |  |
| --- | --- | --- | --- | --- | --- | --- | --- |
|  | | Odds ratio | p | Odds ratio | p | p |  |
| Study: ANSYSCAP | | 0.7 (0.5-0.9) | 0.010 | Ref | - | 0.077 |  |
| Study: TACNOS | |  |  | 0.4 (0.3-0.6) | <0.001 |  |  |
| Study: UCC | |  |  | 0.7 (0.5-1.1) | 0.74 |  |  |
| Age (per 10-year increment) | | 0.7 (0.5-0.9) | 0.015 | 0.6 (0.5-0.8) | <0.001 | 0.73 |  |
| Atrial fibrillation | | 0.7 (0.5-1.0) | 0.028 | 0.5 (0.3-0.8) | 0.003 | 0.29 |  |
| Diabetes | | 0.7 (0.5-0.9) | 0.015 | 0.6 (0.4-0.8) | <0.001 | 0.72 |  |
| Hypertension | | 0.7 (0.5-1.0) | 0.022 | 0.4 (0.2-0.8) | 0.006 | 0.67 |  |
| Referred from other hospital | | 0.7 (0.5-1.0) | 0.039 | 1.8 (1.3-2.6) | <0.001 | 0.46 |  |
| Degree of ipsilateral stenosis | 50-69% conventional^c^ | 0.8 (0.6-1.1) | 0.22 | Ref | - | 0.10 |  |
|  | ≥70% conventional^c^ |  |  | 2.9 (2.1-4.2) | <0.001 |  |  |
|  | CNO without full collapse^c^ |  |  | 2.2 (1.4-3.4) | <0.001 |  |  |
|  | CNO with full collapse^c^ |  |  | 0.3 (0.2-0.5) | <0.001 |  |  |
| Type of presenting event | AFX | 0.7 (0.5-0.9) | 0.017 | Ref | - | 0.90 |  |
|  | RAO |  |  | 0.8 (0.4-1.8) | 0.59 |  |  |
|  | TIA |  |  | 0.7 (0.4-1.1) | 0.12 |  |  |
|  | Stroke |  |  | 0.4 (0.3-0.7) | <0.001 |  |  |
| AFX: Amaurosis fugax. ANSYSCAP: Additional neurological symptoms before surgery of symptomatic carotid stenosis, a prospective study. CNO: Carotid near-occlusion. RAO: Retinal artery occlusion. TACNOS: Transatlantic carotid near-occlusion study. TIA: Transient ischemic attack. UCC: Umeå Carotid Cohort.  ^a^ Included due to association (p<0.1) seen in baseline assessment.  ^b^ Not p<0.1 in baseline (association with sex) and/or bivariable analysis.  ^c^ Includes all cases (regardless of modality). If the model was limited to cases with CTA data, adjusted OR for sex was virtually unchanged at 0.9 (95%CI 0.6-1.3, p=0.47), and degrees of stenosis were similarly virtually unchanged. | | | | | | | |

**Supplemental figure 1.** Kaplan-Meier analyses of the preoperative risk of recurrent ipsilateral ischemic stroke or retinal artery occlusion, stratified by the degree of stenosis. (A) 50-69% conventional. (B) ≥70% conventional. (C) CNO without full collapse. (D) CNO with full collapse.
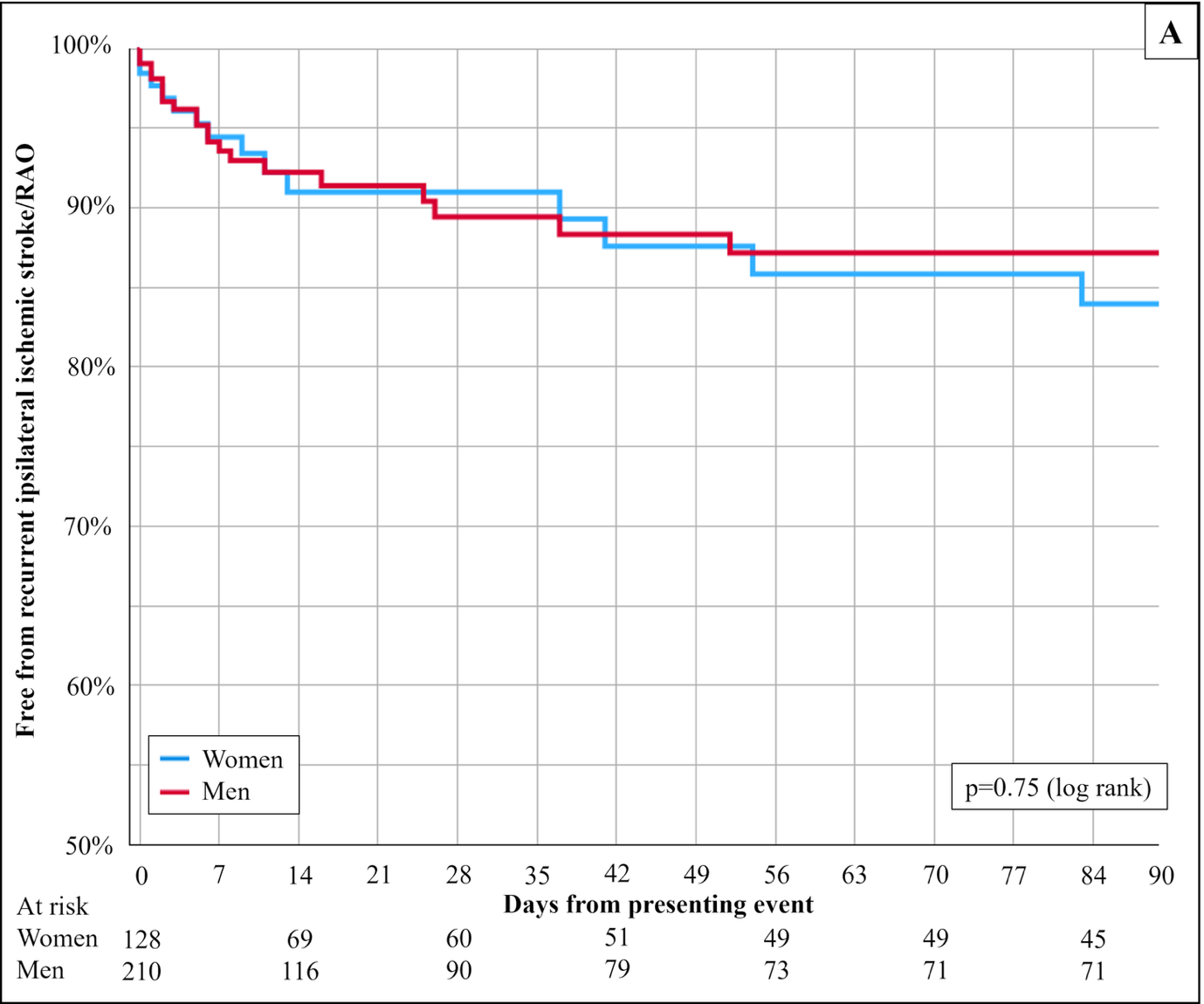

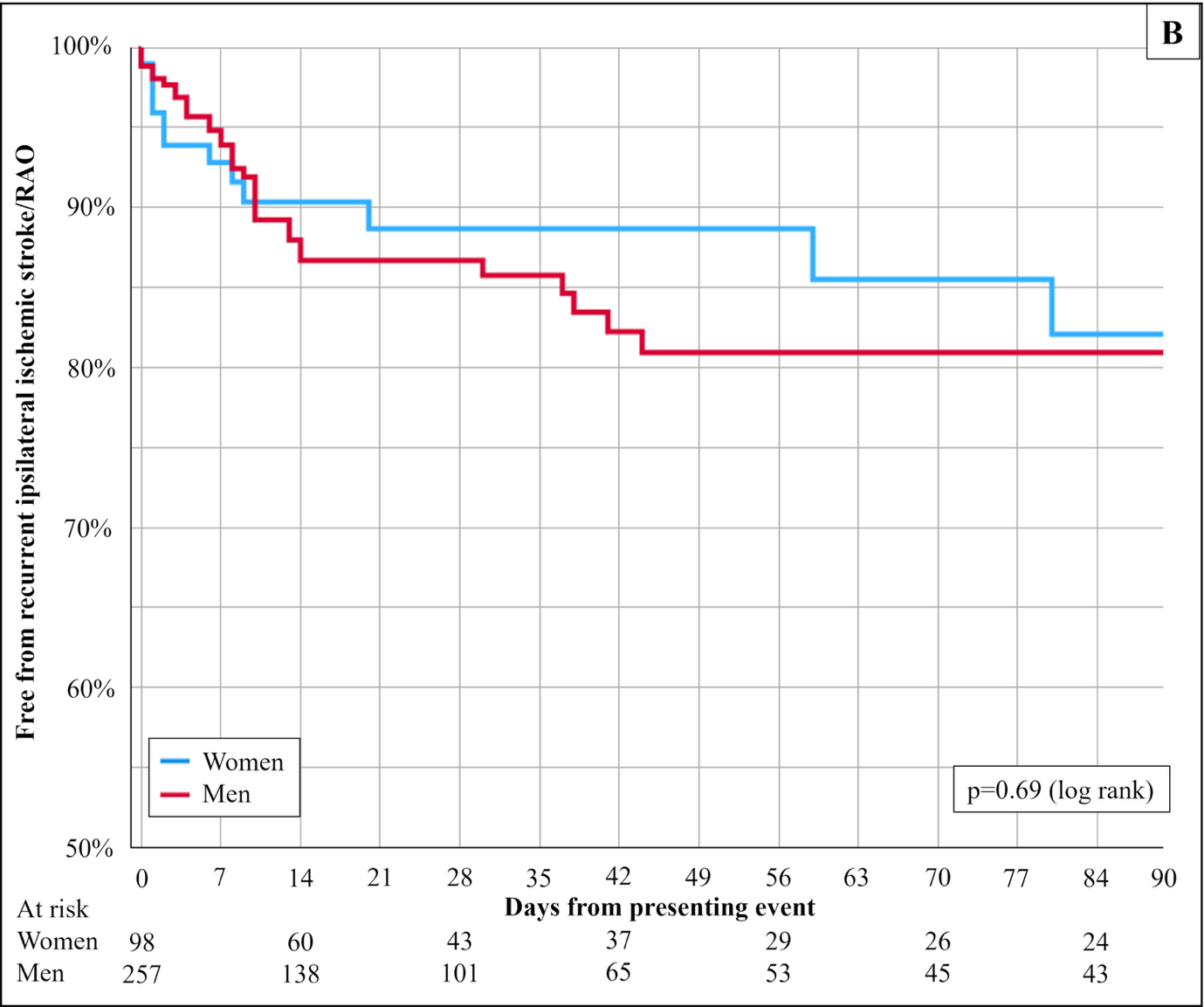

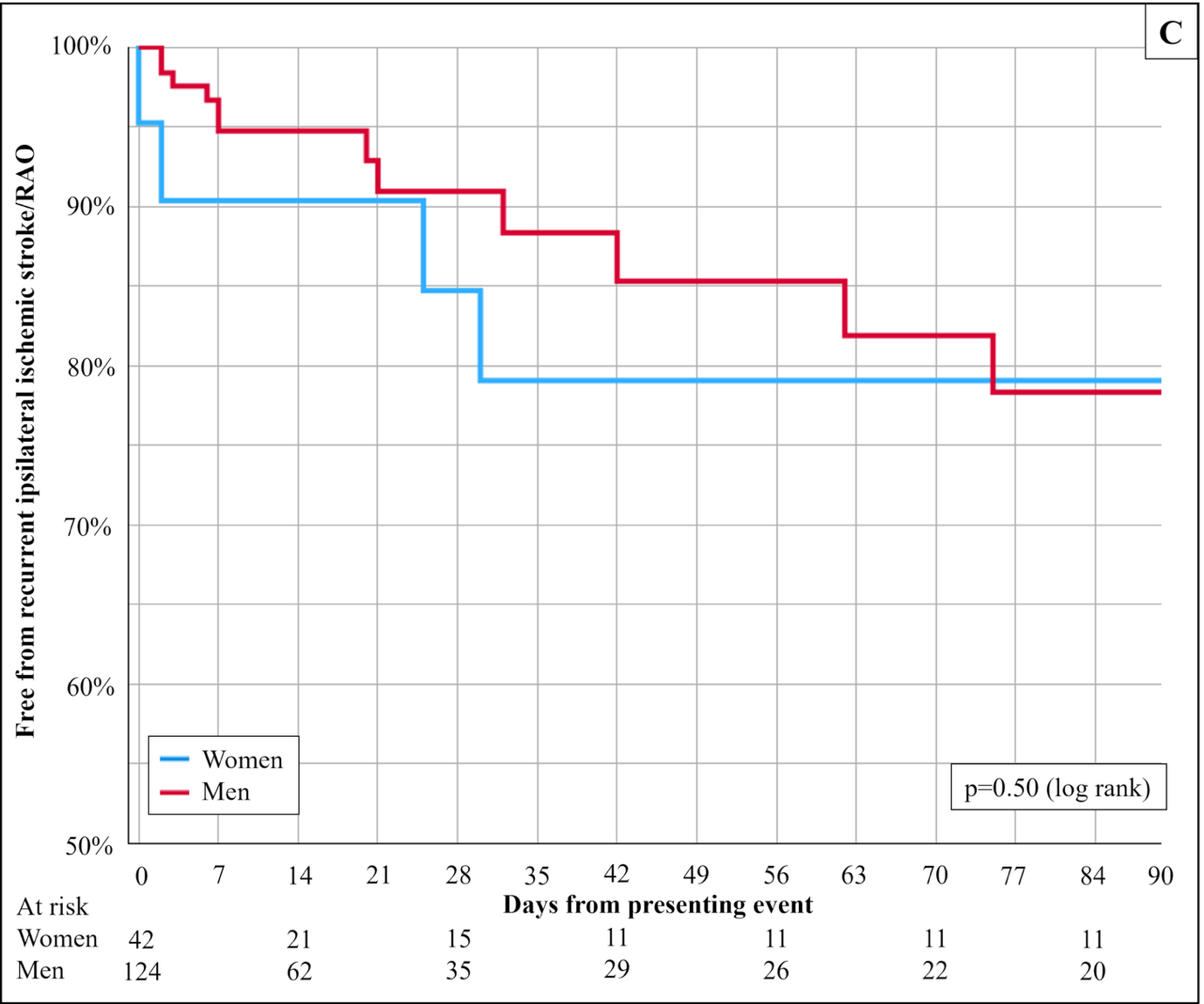

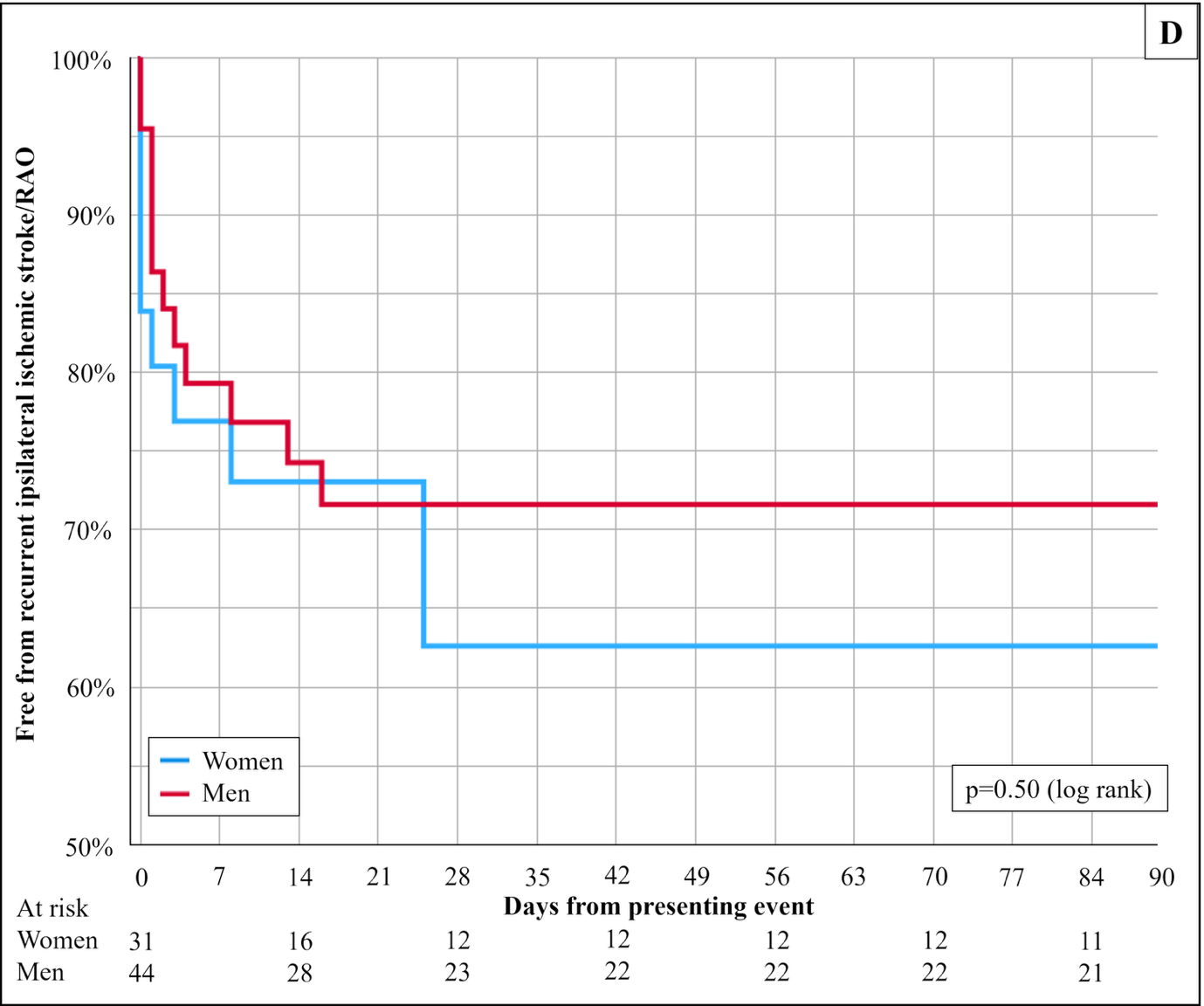


**Supplemental figure 2.** Kaplan-Meier analyses of the preoperative risk of recurrent ipsilateral ischemic stroke or retinal artery occlusion, stratified by the type of presenting event. (A) Cerebral presenting events (stroke or TIA). (B) Retinal presenting event (amaurosis fugax or retinal artery occlusion).


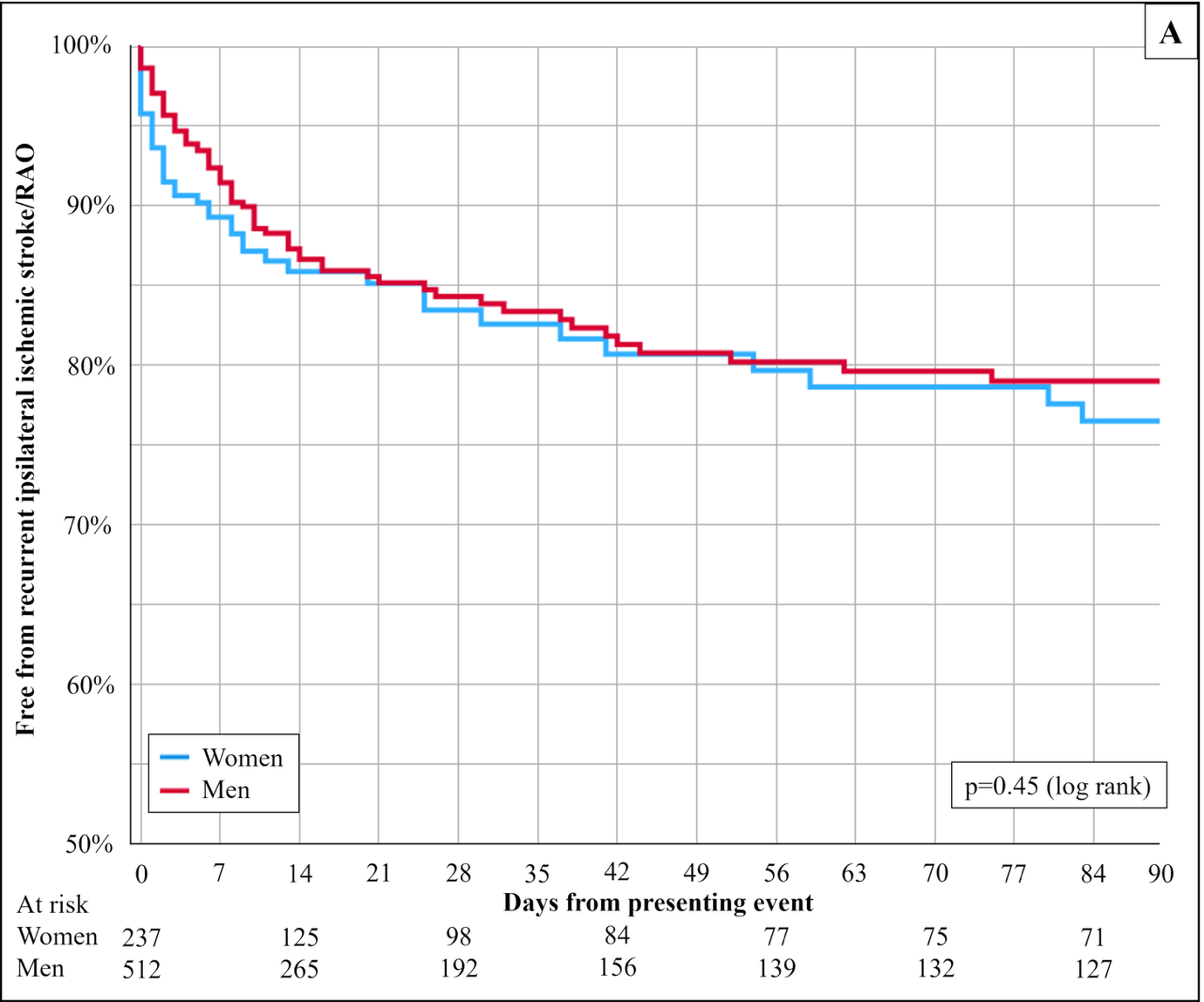


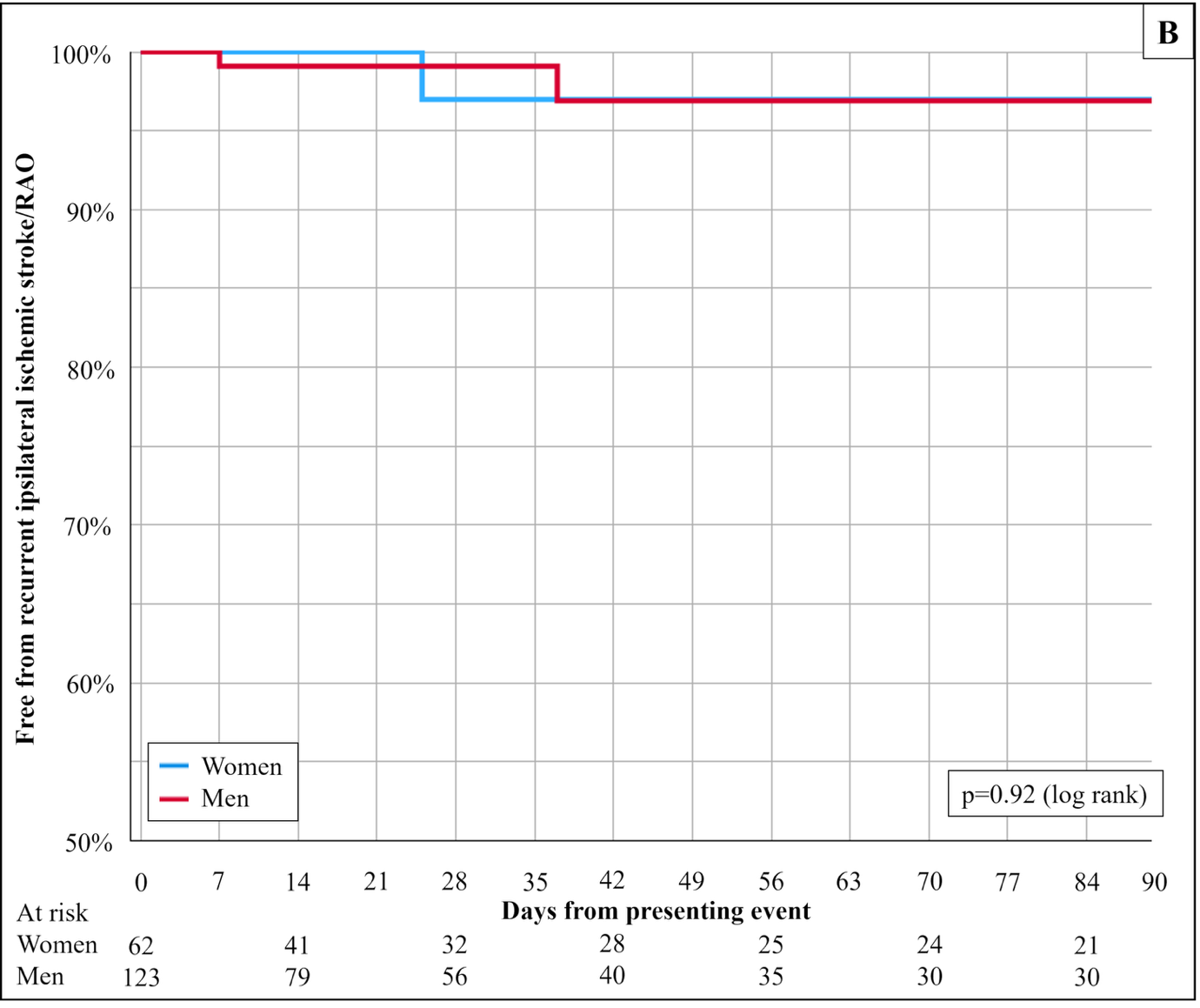

Supplement: aakag018_supplementary [file aakag018_supplementary.zip › aakag018_supplementary/SupplementalfileKremercorr.docx]
